# Editorial: Exercise as a Countermeasure to Human Aging

**DOI:** 10.3389/fphys.2020.00883

**Published:** 2020-07-28

**Authors:** Bradley T. Elliott, Lawrence D. Hayes, David C. Hughes, Martin Burtscher

**Affiliations:** ^1^Translational Physiology Research Group, University of Westminster, London, United Kingdom; ^2^School of Health and Life Sciences, University of the West of Scotland, Glasgow, United Kingdom; ^3^Division of Endocrinology and Metabolism, Department of Internal Medicine, Carver College of Medicine, University of Iowa, Iowa City, IA, United States; ^4^Department of Sport Science, Medical Section, University of Innsbruck, Innsbruck, Austria

**Keywords:** exercise, physical activity, aging, lifespan, healthspan

Unlike many of the branches of natural sciences, there are few true “laws” of physiology. However, there are intrinsic theories about which we are reasonably certain. For example, it is a reasonable statement that exercise and physical activity, in all their forms, typically have positive effects on health and wider physiological function via multiple complex and interacting mechanisms (that we have not yet completely defined). Alternatively, the continuous process of human aging in the adult involves a gradual decline of physiological function across most tissues and systems, again in a complex and intertwining manner. At a point where the average age of humanity is greater than it has ever been, and is continuing to increase, we considered it timely to examine the crossover between these two interacting fields of physiology. Indeed, where past successes in physiology research have emerged from research on transmittable diseases, vaccinations and preventive medicines, our current approaches must now focus on non-transmittable disorders, including frailty syndromes, sarcopenia, and chronic conditions that associate with aging, including heart disease, neuro-cognitive disorders, and diabetes.

When we made this call for submissions, we did not expect the volume of responses we received. In these papers we presented a Research Topic of over 30 articles that covered the interplay between exercise and aging, utilizing approaches that spanned molecular, physiological, and population scale approaches, in both healthy older populations and certain disease subsets, and spanned three *Frontiers* journals *(Frontiers in Physiology, Frontiers in Sports and Active Living, and Frontiers in Aging Neuroscience*). It is a pleasure to note this range of fields and methodological approaches that authors have used.

It has long been known that exercise benefits human function, and that this effect may promote good health into older age. Philostratus (c. 170–250) wrote of individuals who exercised into older age that “They were healthy and did not get sick easily. They stayed youthful into old age, and competed in many Olympics, some in eight and others in nine” (Gymnasticus, p. 44). Several papers in this Research Topic examined classical exercise physiology approaches of a short-term training programme over weeks-to-months. In this vein Kirk et al. gave preliminary results from the LHU-SAT trial, examining 16 weeks of training with or without protein supplementation in healthy over 60-year-old participants. While both groups improved with training, results suggested the protein supplementation group did not improve to a greater degree than the no protein group. However, compliance to protein supplementation beverages in this population continued to be low, an area that may need attention. In line with these results, positive outcomes from classical exercise physiology training interventions were seen by Walker et al. who reported on improved intermuscular coherence, Gavin et al. who noted resistance training improved stair climbing biomechanics in older individuals, Tam et al. reported on resistance training improving exercise economy, and Saeidi et al. findings that a proposed antioxidant altered resistance training-induced changes in circulating adipokines in postmenopausal women. Two exercise physiology interventional papers of note include Franchi et al., who used a novel trampoline plyometric training model in a safe and highly effective way, and Jabbour and Majed with the important observation that the widely used ratings of perceived exertion (RPE) scale over-estimated exercise intensity in sedentary older adults. In meta-analyses reviewing exercise changes from short-term training interventions, endurance exercise decreased pro-inflammatory cytokines concentrations (Zheng et al.), yet counterintuitively testosterone was not improved following training studies in older men (Hayes and Elliott), suggesting resistance training-induced benefits were not via circulating testosterone concentrations.

Updating us on recent advances in targeting mitochondria to offset sarcopenia, Coen et al. reviewed exercise and mitochondrial health for successful aging, reminding the reader that exercise is (for now) the only effective option for treatment of sarcopenia. Linking well to this review, Ubaida-Mohien et al. reported on a proteomic analysis of muscle biopsies from 60 individuals spanning 20–87 years of age, and reported physical activity associated with alterations in proteins governing mitochondria energetics, muscle function, gene health, immunity and senescence, and these changes typically opposed those seen with aging. Mirroring these results, ambulatory older individuals presented a preservation in portions of the myostatin and IGF-I signaling pathways, as well as myocyte structures, that wheelchair bound older individuals did not show (Naro et al.). Differing endurance exercise stimuli improved markers of t-cell senescence (Philippe et al.), while in older rats, muscle protein synthesis responses were blunted relative to younger animals (West et al.). All these results point to an environment that is capable of positively responding to anabolic stimuli, but perhaps not as well as younger muscle tissue, as well as a need for research to separate effects of aging and inactivity.

From a population health point of view, increased lifelong activity, not just short-term exercise interventions, are needed. Thus, there has been much recent interest in examining highly trained masters athletes, as a physiological model of successful aging (Pollock et al., 2015; Elliott et al., 2017). This Research Topic included five reports on lifelong exerciser cohorts. Mancini et al. compared lifelong football players with age matched controls, noting a positive influence of lifelong exercise on markers of auto-lysosomal and proteasomal-mediated processes, while Piasecki M. et al. noted an interesting compensatory mechanism whereby power trained older adults showed increased motor unit size, possibly to compensate for decreased motor unit number. In older females, osteoporosis is often seen, however Onambele-Pearson et al. observes that simple mechanical loading is not sufficient to explain bone density, and that fuller measures of activity and inactivity should be considered. In masters athletes who were grouped as “early” or “late” starters to masters athletics (either lifelong training history or beginning after 50 years of age), Piasecki J. et al. reported no major differences in body composition or bone density between these early and late starters, but both groups reliably demonstrated a healthier phenotype vs. inactive controls. Finally, it was of interest to note positive emotional and cognitive effects of lifelong Tai Chi participation relative to an age-matched control group, which was paired with resting-state fMRI connectively differences (Liu et al.). It can be seen that lifelong activity promotes multiple physiological benefits in an aging population.

At one end of the population size spectrum, Knechtle et al. presented a case study on physiological responses in a 95-year-old masters athlete during a 12 h ultra-marathon event. At the other end are population scale studies. It is of interest to note differences in the association between physical activity, as measured by accelerometery, and relative telomere lengths, with positive associations seen in men but not in woman, across a population of 700 older participants (Stenbäck et al.). By analyzing records of ~27,000 track and field athletes, Ganse et al. observed decreases across maximal power, strength, and endurance records throughout adult lifespan. Further, these declines in performance accelerated post 70 years of age, an observation that was seen in grip strength in the general population (Dodds et al., 2014), and occurred despite high levels of physical activity. These results, in combination, suggested that muscle function loss with age is not only inactivity-induced but has an intrinsic component.

As aging is associated with an increased risk of cardiovascular disease, diabetes and certain types of cancers, and chronic exercise associates with reduced rates of such disorders, it is important to examine exercise in such older populations with such conditions. Indeed, regular exercise training of any type improved quality of life, aerobic capacity and heart function in older heart failure patients (Slimani et al.). Mcleod et al. argued for alterations in guidelines for exercise in the prevention of chronic disorders, promoting the role of resistance training in preventive medicine, interesting reading when paired with the Campbell et al. meta-analysis which observed insufficient evidence to recommend aerobic exercise for vascular function improvement in older sedentary adults. In rats, experimental data suggested that prior exercise training improved survivability from experimental coronary artery occlusion (Veiga et al.), providing us humans with more motivation for maintaining lifelong exercise. This was reinforced by a cohort study of ~3,700 individuals, where both physical activity and sedentary time both independently predicted mortality rates associated with pro-inflammatory conditions (Cabanas-Sánchez et al.). Other findings suggested the improvements in post-exercise reaction time were not different between hypertensive and non-hypertensive patients (Lefferts et al.), and the interesting observation that structural differences in skeletal muscle may underlie difference in stretch shorten cycle between COPD patients and healthy age-matched controls (Navarro-Cruz et al.). These results reinforce the recent American Medical Association's guidelines promoting exercise wherever possible in chronic conditions (Piercy et al., 2018).

Historically, physiology research has primarily utilized the “healthy young male” population, thus we are pleased to note that 14 of the 21 primary experimental papers presented here in human participants included male and female groups, while one specifically examined post-menopausal changes in women. Likewise, we feel the papers presented here give valuable insight concerning the range of aging physiology, in a continuous rather than dichotomous manner. For example, Knechtle et al. concerned a 95-year-old masters athlete, considered the 'oldest old', whereas some papers (Hayes and Elliott) had a minimum age of 60, considered the 'young old'. Moreover, several investigations utilized a young comparison group or a cross sectional design, which permitted authors to study life course aging utilizing multiple research designs.

Both physical activity and structured exercise are near-uniformly positive for human longevity and well-being by multiple, complex physiological mechanisms and pathways that help maintain health, independence and quality of life. Indeed, the complexity of the aging process and the role of exercise in aging physiology were well-represented by the diversity of experimental approaches witnessed in this Research Topic. Combined, the results of these investigations suggested that exercise and activity can offset decreases in human function that we consider “inevitable aspects of aging” but cannot prevent them completely. Our understanding of how and why exercise and activity promote healthy aging, and indeed the basic physiology of the aging process, is currently incomplete. It is our aim that this Research Topic makes a small contribution to the understanding of this complex field.

## Author Contributions

BE wrote the first draft. LH, DH, and MB critically reviewed, and all authors approved the final version of this editorial.

## Conflict of Interest

The authors declare that the research was conducted in the absence of any commercial or financial relationships that could be construed as a potential conflict of interest.
